# P2X7 receptor activation regulates rapid unconventional export of transglutaminase-2

**DOI:** 10.1242/jcs.175968

**Published:** 2015-12-15

**Authors:** Magdalena Adamczyk, Rhiannon Griffiths, Sharon Dewitt, Vera Knäuper, Daniel Aeschlimann

**Affiliations:** Matrix Biology & Tissue Repair Research Unit andArthritis Research UK Biomechanics and Bioengineering Center of Excellence, College of Biomedical and Life Sciences, Cardiff University, Cardiff CF14 4XY, UK

**Keywords:** Transglutaminase, Extracellular matrix stabilization, Purinergic signaling, P2X7 receptor, Unconventional protein secretion, Innate immunity

## Abstract

Transglutaminases (denoted TG or TGM) are externalized from cells via an unknown unconventional secretory pathway. Here, we show for the first time that purinergic signaling regulates active secretion of TG2 (also known as TGM2), an enzyme with a pivotal role in stabilizing extracellular matrices and modulating cell–matrix interactions in tissue repair. Extracellular ATP promotes TG2 secretion by macrophages, and this can be blocked by a selective antagonist against the purinergic receptor P2X7 (P2X7R, also known as P2RX7). Introduction of functional P2X7R into HEK293 cells is sufficient to confer rapid, regulated TG2 export. By employing pharmacological agents, TG2 release could be separated from P2X7R-mediated microvesicle shedding. Neither Ca^2+^ signaling alone nor membrane depolarization triggered TG2 secretion, which occurred only upon receptor membrane pore formation and without pannexin channel involvement. A gain-of-function mutation in P2X7R associated with autoimmune disease caused enhanced TG2 externalization from cells, and this correlated with increased pore activity. These results provide a mechanistic explanation for a link between active TG2 secretion and inflammatory responses, and aberrant enhanced TG2 activity in certain autoimmune conditions.

## Abstract (precis)

**Summary:** Purinergic signaling regulates unconventional secretion of transglutaminase-2 (TG2) and explains the link between aberrant protein modifications and inflammatory responses in TG2-dependent autoimmunity.

## INTRODUCTION

Unconventional export of cytoplasmic proteins [i.e. the processes by which proteins that do not follow the classical endoplasmic reticulum (ER)-to-Golgi secretory pathway are secreted by cells] is being studied extensively because many molecules that fall into this category constitute potent biological signals with key roles in developmental or inflammatory processes. Such proteins lack posttranslational modifications that occur during ER-to-Golgi protein maturation but might be subject to *N*-terminal processing and acetylation or acylation (Muesch et al., 1990; Stegmayer et al., 2005). Several fundamentally different mechanisms appear to support unconventional protein secretion, including self-sustained or transporter-facilitated direct membrane translocation at the plasma membrane, or release in specialized vesicles, the biogenesis of which is distinct from vesicles coated with coat protein complex II (Nickel and Rabouille, 2009; Rabouille et al., 2012). Neither export through the compartment for unconventional protein secretion (CUPS) and multivesicular body pathway nor direct microvesicle shedding at the plasma membrane requires membrane translocation of the cargo, and release is thought to occur by vesicle lysis in the extracellular environment.

Transglutaminases (denoted TG or TGM) are a family of structurally similar enzymes that posttranslationally modify proteins through transamidation, deamidation or esterification of glutaminyl residues (Aeschlimann and Thomazy, 2000). Several of these enzymes have well-established functions in stabilizing extracellular protein assemblies, including TG2 (wound healing), TG4 (semen coagulation) and factor XIII (blood coagulation) (Aeschlimann and Paulsson, 1994; Lorand and Graham, 2003). More recently, TG3 and TG6 have been implicated in extracelluar functions (Zone et al., 2011; Thomas et al., 2013). Despite it being 20 years since we first postulated export of TGs through an unconventional secretory pathway (Aeschlimann and Paulsson, 1994), the underlying process remains elusive. This has gained much attention recently because although matrix stabilization by TG2 is required for an effective tissue repair response, aberrant TG2 action has a central role in the pathogenesis of inflammatory diseases and autoimmunity, most noteably celiac disease (Aeschlimann and Thomazy, 2000; Iismaa et al., 2009). Externalization from cells appears to control TG2 function because Ca^2+^ binding serves as a molecular ‘switch’ for its activation, facilitating transition into a conformation that enables catalysis (Pinkas et al., 2007). Early studies pointed to passive release of TG2 through cell damage (Upchurch et al., 1987; Siegel et al., 2008). More recently, several alternative mechanisms for constitutive release of TG2 have been proposed, including microvesicle shedding (Antonyak et al., 2011; van den Akker et al., 2011) and perinuclear import into Rab11-positive recycling endosomes (Zemskov et al., 2011). However, the proposed mechanisms implicated different domains of TG2 (Chou et al., 2011; Zemskov et al., 2011). Furthermore, constitutive export is difficult to reconcile with the lack of a correlation between TG2 synthesis level and extracellular activity, and the fact that export appears to be cell-type- or differentiation-stage-specific, as exemplified in endochondral bone formation (Aeschlimann et al., 1995). Such sudden, context-dependent externalization of TG2 indicates that its export is regulated by an unidentified signaling event.

One emerging pathway for non-classically secreted proteins including interleukin (IL)-1β involves activation of the purinergic receptor P2X7 (P2X7R, also known as P2RX7), leading to formation of an inflammasome in a NALP3-dependent manner (Dubyak, 2012; Strowig et al., 2012). Inflammasome assembly drives caspase-1 autoprocessing, maturation of IL-1β by caspase-1 cleavage and ultimately IL-1β release (Mariathasan et al., 2006). Activated macrophages derived from P2X7R^−/−^ mice are unable to secrete the mature form of IL-1 family cytokines, including IL-1β and IL-18 (Solle et al., 2001; Pelegrin et al., 2008) and hence, these animals show reduced severity in models of acute inflammatory joint or lung disease (Labasi et al., 2002; Lucattelli et al., 2011; Bartlett et al., 2014).

P2X7R is a member of the P2X family of nucleotide-gated ion channels that is activated by high concentrations of extracellular ATP. Besides K^+^ efflux that triggers inflammasome assembly, the ion channel also supports Ca^2+^ and Na^+^ influx, leading to membrane depolarization and activation of intracellular signaling cascades (Coddou et al., 2011; Bartlett et al., 2014). The P2X4R (also known as P2RX4) crystal structure confirmed that assembly of three subunits, each harboring two transmembrane domains, forms the functional P2X receptor (Kawate et al., 2009). The large extracellular domain has ATP- and metal-ion-binding sites that regulate receptor activation state. Channel opening is associated with conformational changes that reposition the transmembrane segments whereby different states of dilation might be adopted (Hattori and Gouaux, 2012; Jiang et al., 2013). The feature that distinguishes P2X7R from the other P2X family members is a long C-terminal tail (Surprenant et al., 1996; Rassendren et al., 1997) that has been implicated in the process of ‘membrane pore’ formation, which enables plasma membrane permeability to larger organic cations (Virginio et al., 1999; Browne et al., 2013).

High extracellular ATP is a consequence of cell damage, and enforced by ATP release from activated innate immune cells. This acts as a danger signal amplification system that spreads the alarm within the local milieu. However, ATP is not only released upon tissue or cell injury, or stress, but can also be secreted through membrane channels or secretory vesicles (Garcia and Knight, 2010; Sorge et al., 2012; Burnstock, 2015). Given that TG2 is abundantly secreted in the context of inflammation but that extracellular TG2 also has formative roles in tissue development and homeostasis, we hypothesized that its export might be associated with P2X7R signaling. Here, we show for the first time that rapid TG2 export is regulated by P2X7R-mediated membrane pore formation.

## RESULTS

### Macrophages secrete TG2 in a P2X7R-dependent manner

**Fig. 1.**
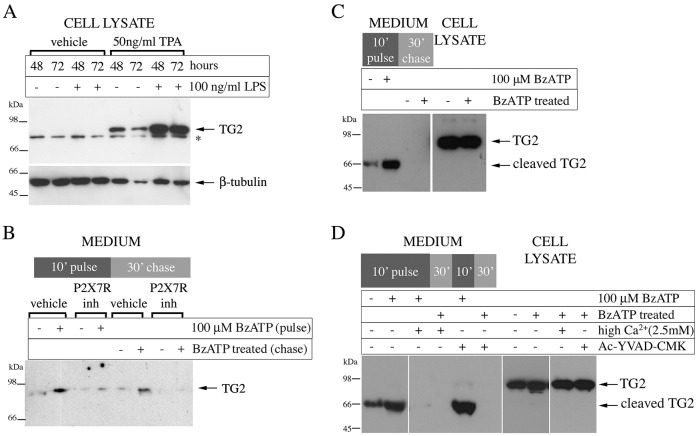
**P2X7R inhibition blocks TG2 secretion by macrophages.** (A) Differentiated monocytes express TG2. THP-1 cells were differentiated for the indicated time with TPA and stimulated with LPS as indicated. Cell extracts were analyzed by western blotting for TG2 or β-tubulin as a loading control (*, non-specific reactivity). (B) TG2 export requires P2X7R activity. Differentiated THP-1 cells were pre-treated with vehicle or 5 μM P2X7R inhibitor A740003 for 10 min, then stimulated as indicated with BzATP for 10 min with or without inhibitor (inh, pulse). Cells were chased for 30 min in P2X7R agonist and antagonist-free medium. Collected medium from the pulse and chase (200 µl) were rendered cell-free by centrifugation, and analyzed for TG2 by western blotting. (C,D) P2X7R activation triggers TG2 secretion in macrophages derived from peripheral blood mononuclear cells. Macrophages were stimulated with BzATP and chased as in B, and collected medium from the of pulse and chase were analyzed for TG2 by western blotting alongside the cell lysates (C). The presence of 100 µM Ac-YVAD-CMK did not prevent externalization or cleavage of TG2, indicating a caspase-1-independent process (D).

The THP-1 monocyte or macrophage cell model was chosen to investigate TG2 export as these cells have been reported to be competent in P2X7R-mediated IL-1β secretion (Mackenzie et al., 2001). We confirmed initially that activation of inflammasome formation by priming cells with lipopolysaccharide (LPS) for Toll-like receptor (TLR) signaling combined with subsequent stimulation with ATP induces IL-1β secretion into the cell supernatant, as determined by capture ELISA (Fig. S1A). TG2 is expressed in differentiated macrophages but not in monocyte precursors (Mehta and Lopez-Berestein, 1986). Therefore, THP-1 cells were treated with the phorbol ester TPA to induce differentiation (Fig. S1B), and TG2 upregulation was confirmed by western blotting of cell lysates (Fig. 1A). We then used the ATP analogue BzATP for P2X7R activation as it shows a high degree of selectivity for P2X7R and does not activate P2Y family ATP-sensing receptors (Coddou et al., 2011). Differentiated cells were stimulated with BzATP for 10 min, and culture supernatants were collected at the end of agonist treatment (pulse) and after a further 30 min in the absence of agonist (chase) to capture immediate and potentially delayed TG2 secretion. BzATP stimulation induced a substantial increase in TG2 secretion as determined by western blotting of cell-free supernatant (Fig. 1B). TG2 export was blocked by the P2X7R antagonist A740003 (Fig. 1B), which inhibits IL-1β secretion in differentiated monocytes (Honore et al., 2006). To substantiate this finding, we analyzed TG2 secretion in response to P2X7R activation in primary human M1 macrophages. BzATP triggered rapid TG2 secretion, contributing to soluble (Fig. 1C) and cell-surface-associated enzyme (Fig. S1C), whereby the soluble enzyme was undergoing processing generating a ∼66 kDa species. Processing did not involve inflammasome-associated caspase-1 nor cell surface MT1-MMP (also known as MMP14) cleavage (Belkin et al., 2001) as it occurred in the presence of *N*-acetyl-YVAD-chloromethyl ketone (Ac-YVAD-CMK) and EDTA, respectively (Fig. 1D; Fig. S1C). Collectively, these data show that P2X7R regulates not only 1L-1β but also TG2 secretion in macrophages.

### Expression of P2X7R confers agonist-inducible, rapid TG2 secretion to HEK293 cells

To investigate whether P2X7R alone was sufficient or other inflammasome pathway components are required for TG2 export, we established HEK293 cells stably expressing wild-type (hereafter P2X7R cells) or tagged human P2X7R. This cell model was selected as it lacks endogenous expression of P2X receptor family members (Mackenzie et al., 2005) and secretes mature IL-1β in response to agonist when co-transfected with P2X7R and pro-caspase-1 (Gudipaty et al., 2003). P2X7R expression was confirmed by western blotting of cell extracts, whereby for tagged P2X7R a single band reactive to antibodies against the P2X7R and the V5 tag was detected (Fig. S2A). Immunocytochemistry confirmed membrane localization of the receptor in P2X7R cells and its absence in parental cells (Fig. S2B). In order to assess P2X7R functionality, changes in the intracellular free Ca^2+^ concentration in response to BzATP were investigated using Fluo-4-AM. Only P2X7R cells, and not parental cells, responded to this agonist (Fig. S2C). A dose–response analysis for BzATP stimulation of P2X7R cells using Ca^2+^ signaling as a readout derived an apparent *K*_D_ of ∼75 µM (Fig. S2D). This is in line with literature data ranging from 40–100 µM depending on extracellular Ca^2+^ concentration (Rassendren et al., 1997). Therefore, stimulation with 100 µM BzATP produced a P2X7R-specific and, in terms of ligand occupancy, relevant response for further investigation of downstream events.

**Fig. 2.**
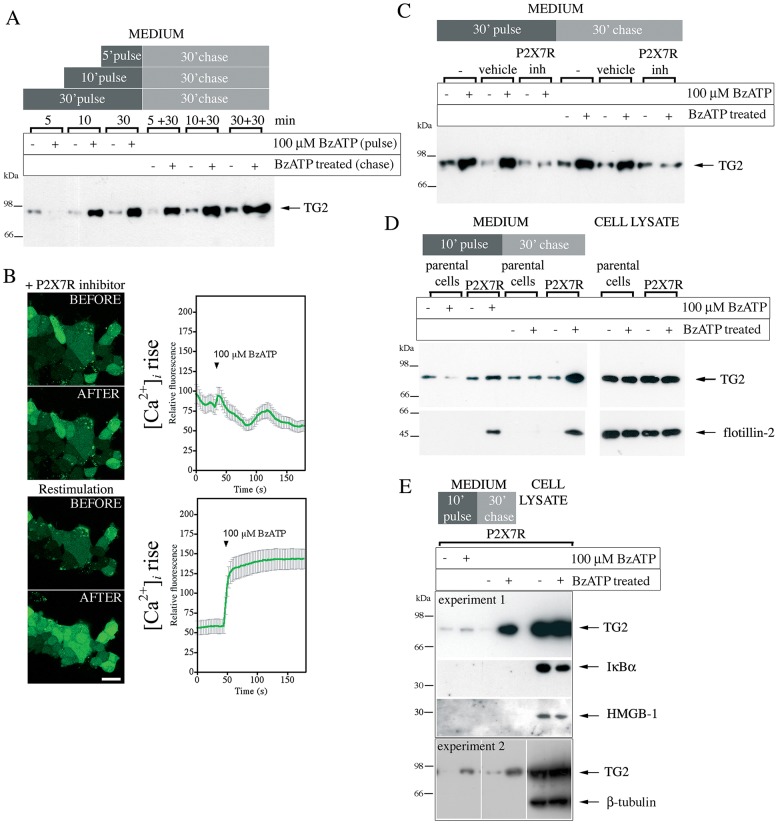
**P2X7R activation mediates TG2 externalization.** (A) Analysis of TG2 secretion in HEK293 P2X7R cells. TG2-transfected cells were stimulated with BzATP or vehicle for indicated time (pulse), then incubated for 30 min in agonist-free medium (chase). TG2 secretion into cell-free supernatants was assessed by western blotting. (B) Inhibitor A740003 reversibly blocks P2X7R activation. P2X7R cells were incubated with Fluo-4-AM and 5 μM P2X7R inhibitor for 20 min prior to BzATP stimulation in the presence of inhibitor (top), washed with inhibitor-free medium for 5 min, and then re-stimulated with BzATP (bottom). The fluorescence (λ_ex_, 488 nm; λ_em_, 500–535 nm) change in individual cells was monitored by confocal microscopy (mean±s.e.m., *n*=30) (right). Optical sections of the same field before and 180 s after BzATP addition are shown (left). Scale bar: 25 µm. (C) P2X7R inhibitor (inh) blocks TG2 secretion. TG2-transfected P2X7R cells were pre-treated with P2X7R inhibitor or vehicle for 10 min before BzATP stimulation as indicated. TG2 release into medium was assessed as in A. (D,E) Cells release membrane-bound particles upon P2X7R activation. TG2 transfected P2X7R or parental cells were BzATP stimulated for 10 min, and chased in agonist-free medium. Conditioned media and cell lysate were analyzed by western blotting for TG2 and the microvesicle marker flotillin-2 (D) or, as a control, β-tubulin, IκBα and HMGB-1 (E).

We then investigated whether P2X7R activation induces TG2 secretion. TG2-transfected P2X7R cells were treated with agonist for 5, 10 or 30 min, followed by a 30-min chase period after agonist wash out. Supernatants of both fractions were analyzed for TG2 by western blotting. Within 10 min of BzATP application, pulse fractions revealed substantial TG2 secretion in agonist-treated but not vehicle-treated cells (Fig. 2A). No TG2 export was seen after 5 min indicating that kinetics were considerably slower than Ca^2+^ signaling. Interestingly, elevated TG2 levels in the chase fraction were observed in cells that were exposed to BzATP for 5 min (Fig. 2A) or even 1 min (data not shown), indicating that P2X7R activation, and not subsequent events occurring upon prolonged agonist exposure, triggers TG2 export. As TG2 levels in the chase fraction were independent of the agonist exposure time (Fig. 2A), it appears that, once initiated, the TG2 export mechanism is active over an extended time period and leads to gradual extracellular TG2 accumulation at a constant rate. Note, the amount of secreted TG2 is small compared to the total and, hence, export does not deplete cellular TG2 over the time period investigated (Fig. 2D, cell lysate). To further demonstrate that this cell response required P2X7R activity, we employed the competitive P2X7R inhibitor A740003. At 5 µM, it completely blocks a rise in the intracellular Ca^2+^ concentration ([Ca^2+^]_i_) in response to BzATP (Fig. 2B, top panel), and this is reversible upon inhibitor wash out (Fig. 2B, bottom panel). BzATP stimulation of cells in the presence of this inhibitor was unable to trigger TG2 secretion (Fig. 2C), demonstrating that active secretion of TG2 is a P2X7R-regulated process.

### P2X7R-mediated TG2 export is not linked to loss of cell membrane integrity or apoptosis

Shedding of membrane-bound particles containing TG2 together with the lipid raft protein flotillin-2 has been reported (Antonyak et al., 2011). Hence, we investigated whether P2X7R-mediated TG2 secretion correlated with flotillin-2 release. Western blot analysis of cell lysates confirmed that flotillin-2 and TG2 were expressed at comparable levels in P2X7R and parental cells (Fig. 2D, cell lysate). Only P2X7R cells responded to BzATP stimulation with release of flotillin-2 into the cell supernatant, indicating P2X7R-dependent vesicle shedding (Fig. 2D, medium). Similar to TG2, flotillin-2 was present in the pulse fraction and accumulated in the chase fraction, potentially indicating co-release. To further analyze secreted material and exclude protein release through passive cell lysis we investigated externalization of the cytosolic proteins IκBα, GAPDH and β-tubulin, as well as of HMGB-1 which is secreted non-classically via the exosome pathway (Lu et al., 2012). We were unable to detect any of these proteins in the cell supernatant after P2X7R activation (Fig. 2E and data not shown). However, given that prolonged stimulation of P2X7R can lead to cell death (Mackenzie et al., 2005), and this crucially affects the conclusions, we designed experiments to more selectively investigate loss of membrane integrity and apoptosis, respectively. First, release of cytosolic lactate dehydrogenase (LDH) was quantified after stimulation of either P2X7R or parental cells with BzATP for 10 min. No P2X7R-induced release of LDH was seen (Fig. S3A). Second, BzATP-treated P2X7R cells were chased for various times up to 22 h and assessed for caspase-3 activation by western blotting. Activated caspase-3 could not be detected at any time (Fig. S3B) whereas within 6 h of TNFα stimulation caspase-3 cleavage was evident as reported (Arlt et al., 2003). These data show that TG2 externalization is not related to cell damage or death but is a selective process, possibly linked to P2X7R-dependent membrane changes. This is consistent with activation of P2X7R triggering rapid alterations in membrane topology without causing cell death in a manner that completely reverses as [Ca^2+^]_i_ falls, unless receptor stimulation is sustained for long time periods (Mackenzie et al., 2005).

### TG2 localizes to membrane subdomains upon cell stimulation with P2X7R agonist

**Fig. 3.**
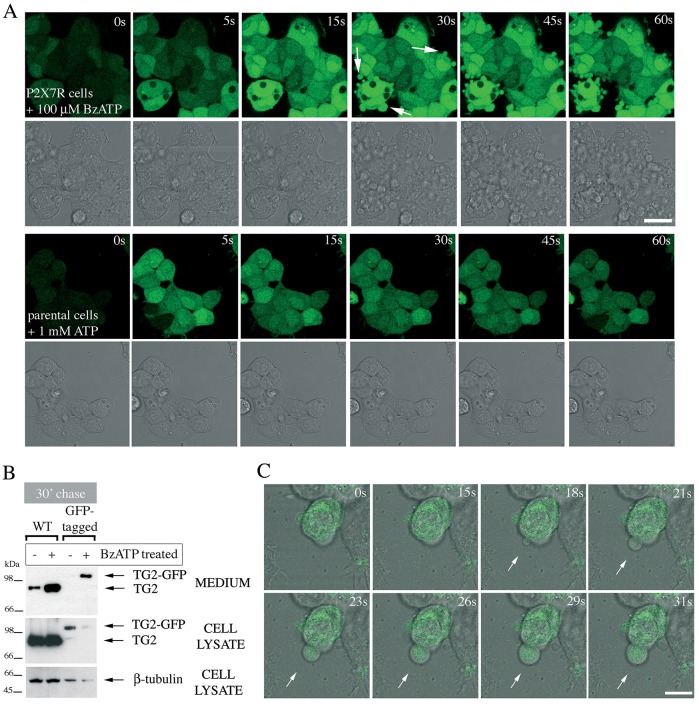
**Membrane blebs induced by P2X7R activation contain TG2.** (A) P2X7R signaling induces rapid membrane blebbing. Fluo-4-AM-loaded P2X7R cells were stimulated with BzATP while acquiring fluorescence and phase-contrast images by real-time microscopy to visualize morphological changes and Ca^2+^ signaling simultaneously (top). Membrane blebs are indicated by arrows. ATP stimulation of parental cells induces oscillating Ca^2+^ signals but no overt morphological changes (bottom). Scale bar: 25 µm. (B,C) TG2 redistributes into membrane blebs. To confirm export of tagged TG2, TG2- (wild-type, WT) or TG2–GFP-expressing P2X7R cells were stimulated with 100 µM BzATP for 10 min, chased for 30 min in agonist-free medium, followed by analysis of conditioned media and cell extracts for TG2 by western blotting (B). To localize GFP-tagged TG2 during BzATP stimulation, real-time confocal microscopy was employed. Genesis of a membrane bleb is depicted (arrows), with an optical section of GFP fluorescence overlaid onto phase-contrast images to correlate morphological changes with changes in TG2 distribution (C). Scale bar: 10 µm.

In P2X7R-expressing cells, the prolonged increase in [Ca^2+^]_i_ upon BzATP application was followed within 30 s by extensive cell blebbing as visualized by real-time microscopy (Fig. 3A, arrows). The term ‘blebbing’ is used here to describe formation of plasma membrane projections due to Rho-dependent actin reorganization that follow P2X7R activation (MacKenzie et al., 2001; Pfeiffer et al., 2004)*.* This response is P2X specific. Stimulation of the parental cells, which express P2Y receptors, with ATP induced smaller transient oscillations in [Ca^2+^]_i_ but no apparent morphological changes (Fig. 3A). This led us to speculate that TG2 externalization might be linked to membrane bleb formation, and we used GFP-tagged TG2 to monitor its redistribution in live cells. We confirmed that P2X7R activation triggered externalization of tagged TG2 similar to wild-type TG2 (Fig. 3B). Analysis by confocal microscopy revealed a clear ubiquitous cytoplasmic distribution for TG2–GFP (Fig. 3C). Upon P2X7R activation, TG2–GFP was rapidly translocated into membrane blebs, and freely re-localized to sites where new membrane protrusions formed (Fig. 3C, arrow). However, despite abundant bleb formation, careful reconstruction from image sequences revealed that these large membrane protrusions remained continuous with the plasma membrane and were eventually retracted by cells. We obtained similar results for N- and C-terminally tagged TG2 indicating that the position of the tag did not substantially alter protein localization. Although we were unable to directly visualize TG2 release, a noticeable reduction in fluorescence upon P2X7R activation indicated that the intracellular pool of TG2 was rapidly diminished, consistent with its relocation into the medium (Fig. 3B).

### P2X7R-agonist-induced TG2 secretion is independent of microvesicle shedding

**Fig. 4.**
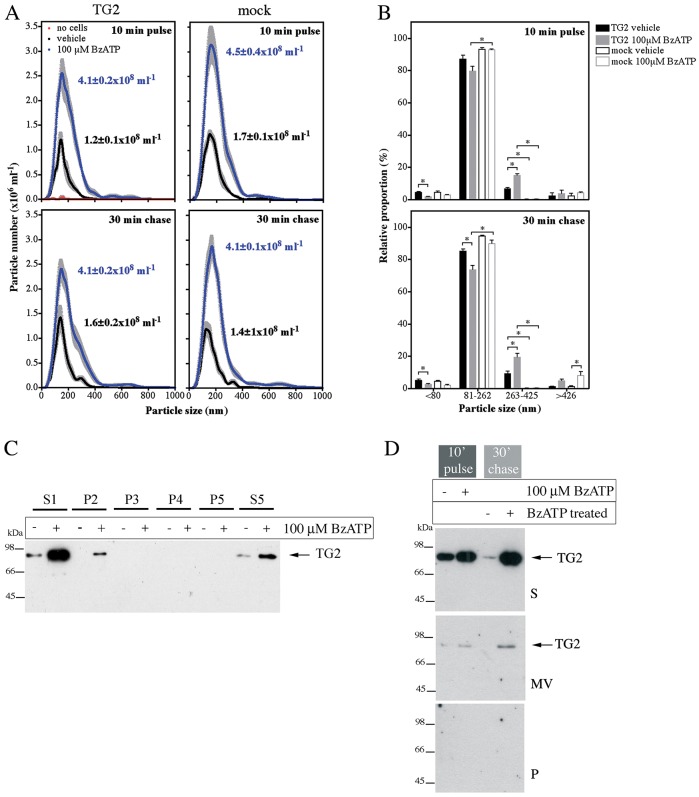
**P2X7R-mediated TG2 export is not due to microvesicle release.** (A,B) Analysis of vesicle release by nanoparticle tracking. TG2- or mock-transfected P2X7R cells were stimulated with BzATP for 10 min, chased for 30 min in agonist-free medium, and conditioned media were analyzed for nanoparticles using light scattering in combination with particle tracking (Nanosight). Particle distribution and total particle concentration is shown (mean±s.e.m.; *n*=5) (A). Particles were broadly assigned to one of four fractions based on volume: representing exosomes (∼60 nm; ≤80 nm diameter), microvesicles (∼145 nm; 81–262 nm), larger vesicles (∼335 nm; 263–425 nm) and aggregates or membrane blebs (≥426 nm) (B). (C,D) Analysis of isolated microvesicles for TG2. Cell-free medium (S1) from BzATP- or control-treated cells was subjected to differential centrifugation (P, pellet; S, supernatant): in C, 3000 ***g*** twice (P2, P3), 10,000 ***g*** (P4), and 100,000 ***g*** (P5, S5), and in D, 3000 ***g*** followed by separation of microvesicles (MV) on a sucrose cushion. Fractions were analyzed for TG2 by western blotting.

As small vesicles might be released by cells that are beyond the resolution of conventional confocal microscopy, we used light scattering combined with particle tracking to further analyze cell-free supernatants for nanoparticles. A robust increase in particle shedding by P2X7R cells upon BzATP treatment was observed during stimulation and in the subsequent chase period (Fig. 4A). Most of the secreted particles had diameters of 81–262 nm (Fig. 4B) in line with more variably sized microvesicles, rather than exosomes that originate from multivesicular bodies, which are size-constrained and typically <90 nm (Cocucci et al., 2009). TG2 expression modestly increased the proportion of larger particles (Fig. 4B) but did not substantially alter total particle release (Fig. 4A). To understand whether TG2 localizes in microvesicles, freshly harvested conditioned medium was subjected to differential centrifugation and resulting pellets and supernatant were analyzed by western blotting (Fig. 4C). TG2 mainly localized to the 100,000 ***g*** supernatant fraction containing soluble proteins (S5), with some TG2 found in very large aggregates or associated with organelles (P2) but not in the microvesicle fraction (P4). To substantiate this, microvesicles were separated using a sucrose density gradient (Fig. 4D). Again, TG2 was predominantly in the soluble protein fraction. These data suggest that although P2X7R activation induces abundant microvesicle release by cells, secreted TG2 is not apparently associated with microvesicles but present as a free form.

### Extracellular Ca^2+^ regulates TG2 externalization, but its secretion is independent of catalytic enzyme functions

**Fig. 5.**
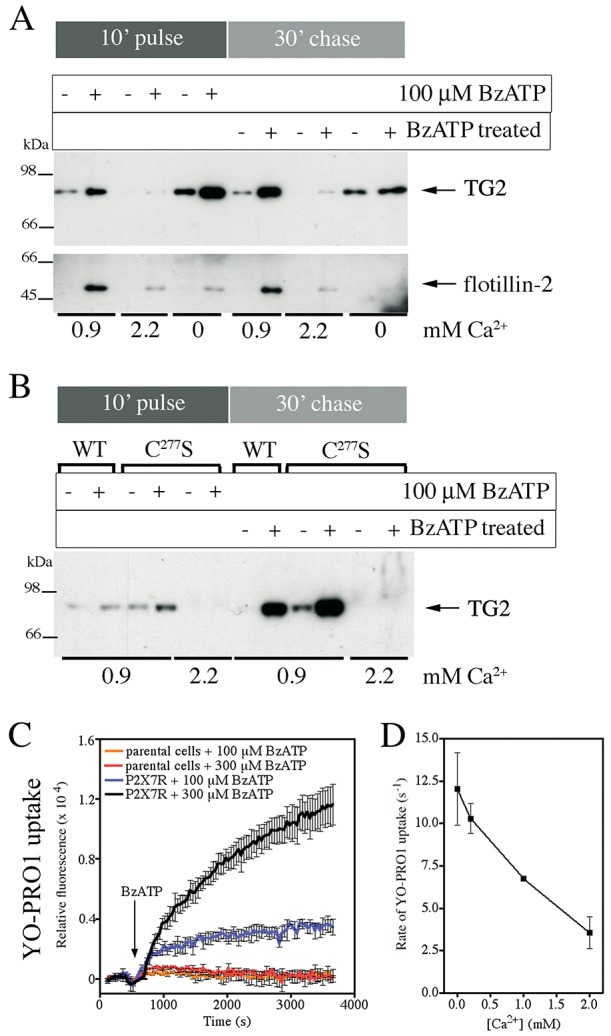
**Extracellular Ca^2+^ regulates TG2 secretion.** (A) P2X7R-mediated TG2 export at different [Ca^2+^]_ex_. P2X7R cells expressing TG2 were stimulated with BzATP for 10 min in medium containing 0.9 or 2.2 mM Ca^2+^ or in Ca^2+^-free medium, and chased for 30 min in respective media without BzATP. Conditioned media were analyzed by western blotting for TG2 and flotillin-2. (B) TG2 catalytic activity is not required for P2X7R-mediated export. P2X7R cells expressing TG2 or the TG2 C277S mutant were stimulated with BzATP in medium containing 0.9 or 2.2 mM Ca^2+^ and TG2 export was assessed as above. (C,D) [Ca^2+^]_ex_ regulates P2X7R activity. P2X7R or parental cells were stimulated with BzATP, as indicated, in PSS containing YO-PRO1 and different concentrations of Ca^2+^. To determine YO-PRO1 uptake by cells after BzATP application, changes in well-specific fluorescence (λ_ex_, 480-10 nm; λ_em_, 520-10 nm) were monitored over time. A representative experiment of dye uptake in Ca^2+^-free PSS is shown as mean±s.e.m. of two wells (C). In D, the initial rates of YO-PRO1 uptake at different [Ca^2+^]_ex_ in response to 300 μM BzATP are given (mean±s.e.m.; *n*=2).

TG2 secretion was effectively stimulated by P2X7R activation in medium that contains 0.9 mM Ca^2+^, which is similar to the free ionized extracellular Ca^2+^ concentration estimated at 1.1–1.3 mM (Riccardi and Kemp, 2012), but surprisingly not in medium containing high Ca^2+^ (Figs 1D and 5A). BzATP treatment of cells in the absence of Ca^2+^ led to enhanced TG2 secretion during stimulation only (Fig. 5A), indicating that TG2 export was faster but not sustained. In contrast, flotillin-2 release occurring at 0.9 mM Ca^2+^ was greatly reduced when cells were stimulated with agonist at either 0 or 2.2 mM Ca^2+^ (Fig. 5A). This shows that TG2 and flotillin-2 secretion is differentially affected by the extracellular Ca^2+^ concentration ([Ca^2+^]_ex_) and hence, that the underlying mechanisms are distinct. As microvesicle shedding is a Ca^2+^-dependent process, TG2 release in Ca^2+^-free medium supports a vesicle-independent mode of release, in line with previous data (Fig. 4).

To exclude Ca^2+^-dependent loss of externalized TG2 due to cell surface retention through interaction with substrates or autocatalytic crosslinking, we compared secretion of wild-type TG2 with crosslinking-incompetent TG2 C277S (Stephens et al., 2004). BzATP stimulation of cells induced export of both TG2 and TG2 C277S at 0.9 mM Ca^2+^ but not at 2.2 mM (Fig. 5B). This indicates that the lack of TG2 secretion at high [Ca^2+^]_ex_ is not due to TG2 activity but might reflect differences in the P2X7R activation state. This is further supported by high [Ca^2+^]_ex_ also affecting flotillin-2 release (Fig. 5A). Besides cation transport, activation of P2X7R can lead to ‘membrane pore’ formation which manifests as apparent permeability of the plasma membrane to cationic molecules such as YO-PRO1 (Virginio et al., 1999; Pelegrín, 2011; Browne et al., 2013). Measurement of YO-PRO1 uptake confirmed that P2X7R cells, but not parental cells, form membrane pores upon BzATP treatment (Fig. 5C), and that the dye uptake rate is inversely correlated to [Ca^2+^]_ex_ (Fig. 5D). Ca^2+^–nucleotide interactions could potentially limit the effective agonist concentration. However, the observed BzATP dose response is not consistent with this explanation (Fig. S3C). Therefore, in our experiments, Ca^2+^ likely acts as an allosteric regulator of P2X7R, either directly or indirectly inhibiting receptor activation as previously suggested (Yan et al., 2011). Taken together, this suggests that high [Ca^2+^]_ex_ is an important negative regulator of TG2 secretion, whereby Ca^2+^ ions appear to regulate P2X7R activation rather than influencing TG2 activity during export.

### TG2 export is linked to P2X7R-mediated membrane pore formation

**Fig. 6.**
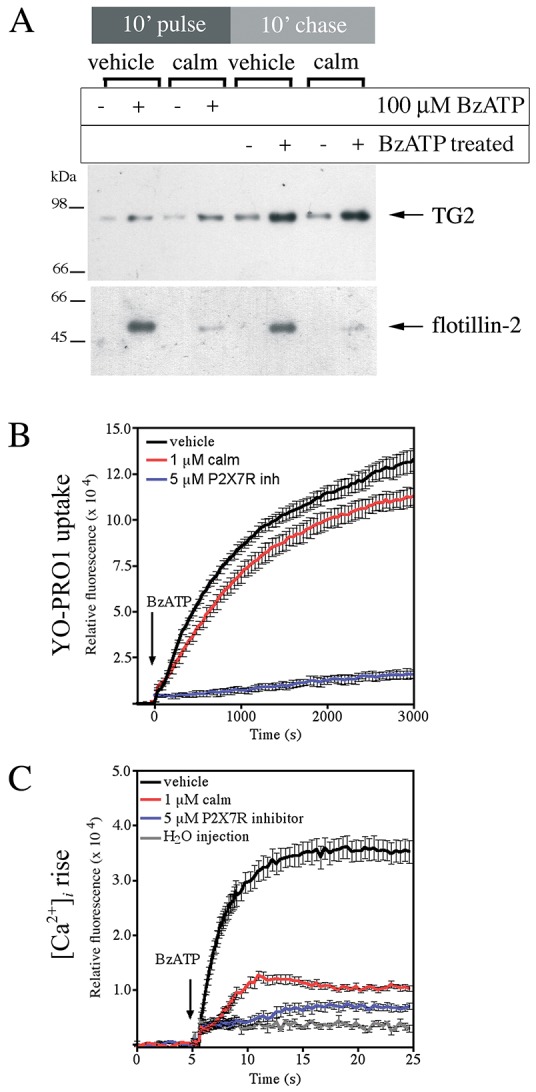
**TG2 export is independent of K^+^ efflux and membrane depolarization.** (A) Calmidazolium (calm) blocks flotillin-2 but not TG2 release. TG2-transfected P2X7R cells were pre-treated for 10 min and then stimulated with BzATP in medium containing 1 µM calmidazolium or vehicle. Cells were chased in agonist-free medium, and conditioned media analyzed by western blotting for TG2 and flotillin-2. (B) Calmidazolium does not affect P2X7R-dependent ‘membrane pore’ formation. P2X7R cells were pre-treated with calmidazolium, P2X7R inhibitor A740003 or vehicle for 10 min prior to stimulation with 100 µM BzATP in the presence of respective inhibitors or carrier in PSS containing YO-PRO1 and 0.9 mM Ca^2+^. Dye uptake was monitored over time. Results are shown as mean±s.e.m. of two wells, and are representative of three independent experiments. (C) Calmidazolium ameliorates the large rise in [Ca^2+^]_i_. Fluo-4-AM-loaded P2X7R cells were pre-treated with calmidazolium, P2X7R inhibitor or vehicle for 20 min prior to stimulation with 100 μM BzATP in the presence of inhibitors or carrier. Fluorescence change (λ_ex_, 485-12 nm; λ_em_, 520-10 nm) relative to control in response to agonist treatment was monitored (mean±s.e.m. of eight replicate wells).

To assess the contribution of the initial ion flux on TG2 secretion, calmidazolium was employed. This compound is an inhibitor with broad selectivity for voltage-gated fast-acting Na^+^/K^+^ and L-type Ca^2+^ channels that also inhibits the initial ATP-evoked ion flux through P2X7R without affecting the downstream membrane pore formation (Virginio et al., 1997). Calmidazolium has an extracellular mode of action on P2X7R. BzATP-induced TG2 export in P2X7R cells was unaffected by the presence of calmidazolium but flotillin-2 secretion was blocked (Fig. 6A). The inhibitor had no effect on pore formation activity of P2X7R (Fig. 6B) but substantially reduced the rise in [Ca^2+^]_i_ mediated by P2X7R activation (Fig. 6C). This indicates that TG2 secretion is linked to P2X7R-dependent pore formation but not the initial ion flux and associated membrane depolarization.

Given that TG2 secretion was induced by P2X7R activation in Ca^2+^-free medium but the kinetics of export were altered (Fig. 5A), we investigated whether Ca^2+^ release from intracellular stores plays a role. P2X7R cells were pre-loaded with the Ca^2+^ chelator BAPTA-AM to buffer free cytosolic Ca^2+^ prior to BzATP stimulation in Ca^2+^-free medium. This reduced TG2 release to near baseline levels (Fig. S3D), confirming that Ca^2+^ signaling has a role in TG2 export as previously suggested (Zemskov et al., 2011). Conversely, cyclopiazonic acid (CPA) was applied to inhibit the SERCA Ca^2+^ transporter to trigger a rise in [Ca^2+^]_i_ in the absence of P2X7R activation. CPA addition alone was unable to induce TG2 secretion (Fig. S3D), despite inducing a peak [Ca^2+^]_i_ of the same magnitude as P2X7R activation when used at 20 µM (Fig. S3E). This indicates that a rise in [Ca^2+^]_i_ by itself is not sufficient to induce TG2 export.

### TG2 secretion is pannexin independent but enhanced by activating mutations in P2X7R

**Fig. 7.**
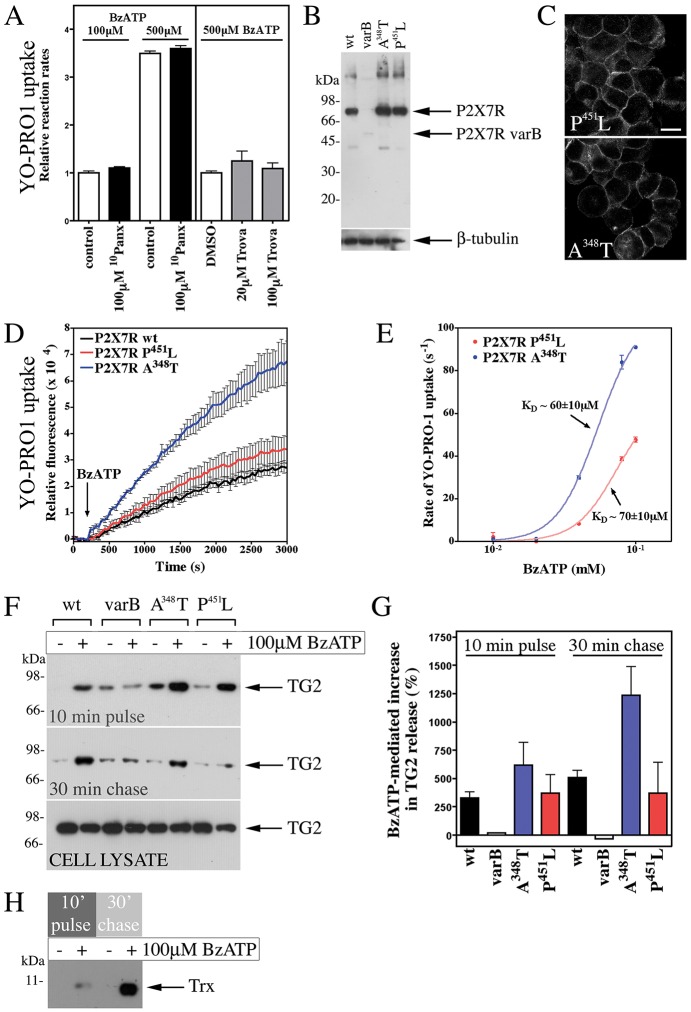
**P2X7R-mediated membrane pore formation is required for TG2 externalization.** (A) P2X7R-mediated pore formation is pannexin independent. P2X7R cells were pre-treated with ^10^Panx or trovafloxacin (Trova) as indicated, and then stimulated with BzATP in PSS with respective inhibitors, YO-PRO1 and 0.9 mM Ca^2+^. Results are given as initial rates of dye uptake relative to control (mean±s.e.m.; *n*=3). Pannexin inhibitors did not affect dye uptake, neither at limiting nor saturating agonist concentration. (B,C) Characterization of expression of mutant P2X7Rs. Extracts of cells stably expressing wild-type (wt), A348T or P451L P2X7R, or the P2X7R variant B (varB) were analyzed by western blotting with antibodies against the P2X7R extracellular domain and β-tubulin, as a loading control (B). Membrane localization of receptor was confirmed by immunocytochemistry (C; compare to Fig. S2B). Images reflect an optical section acquired by confocal microscopy. Scale bar: 12.5 µm. (D,E) Pore formation is enhanced in cells expressing P2X7R A348T. YO-PRO1 uptake following stimulation of cells with 100 µM BzATP is shown as mean±s.e.m. (*n*=3) fluorescence (D). Comparison of initial rate of YO-PRO1 uptake for P2X7R-A348T- and P451L-expressing cells highlights increased pore activity for P2X7R A348T but unchanged ligand regulation (E). Results are mean±s.d. (*n*=2). (F–H) TG2 export correlates with receptor pore activity. TG2-transfected cells expressing P2X7R variants were stimulated with BzATP for 10 min, and chased in agonist-free medium. Conditioned media were analyzed by western blotting for TG2 (F), and results (mean±s.e.m., *n*=3) quantified by densitometry (G). Note, cell lysates confirm comparable TG2 expression levels in different cell lines (F). For thioredoxin-1 (Trx) detection, media (P2X7R cells) were analyzed by western blotting after separation in 16% SDS-PAGE Tricine gels (H).

P2X7R-mediated membrane pore formation has been proposed to relate to P2X7R channel dilation upon saturation of ATP-binding sites, possibly combined with acquisition of additional subunits (Browne et al., 2013) or alternatively, by coupling to another channel (i.e. pannexin-1) (Pelegrin and Surprenant, 2007; Gulbransen et al., 2012). We evaluated the latter by treating cells with pannexin inhibitors. Neither the peptidic competitor ^10^Panx (Pelegrin and Surprenant, 2007) nor trovafloxacin (Poon et al., 2014) had any effect on BzATP-stimulated YO-PRO1 uptake (Fig. 7A) or TG2 export. We therefore sought to clarify whether the C-terminally truncated P2X7R splice variant B that lacks pore forming ability (Adinolfi et al., 2010) supports TG2 secretion. However, expression of this variant after site-specific stable integration or transient transfection was very low as determined by western blotting of cell lysates (Fig. 7B), and we were unable to confirm cell surface localization with antibodies against the P2X7R extracellular domain. Nevertheless, we attempted to confirm agonist-mediated membrane depolarization using the sensitive voltage-sensing FRET probes CC2-DMPE and DiSBAC_2_ (Wolff et al., 2003). Only cells expressing wild-type P2X7R showed membrane channel activity (response ratio for P2X7R, 1.53±0.04 with BzATP, 2.17±0.12 with KCl, 1.07±0.06 with control solution; for P2X7R variant B, 1.10±0.03 with BzATP, 1.74±0.10 with KCl; mean±s.d., *n*=4), suggesting altered trafficking and degradation of the truncated receptor variant.

**Fig. 8.**
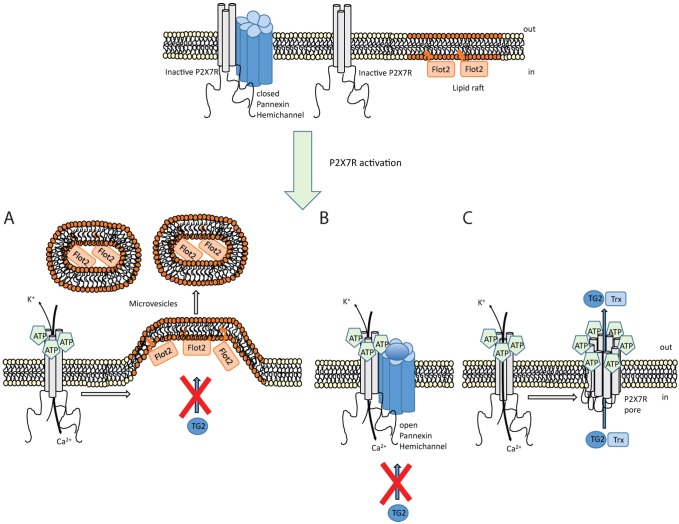
**Mechanism controlling TG2 export.** Schematic showing different events occurring upon P2X7R activation by ATP. (A) Ion channel activity triggers intracellular signaling that results in actin reorganization and microvesicle shedding. However, these microvesicles do not contain TG2. (B) Coupling between P2X7R and pannexin-1 triggers hemichannel pore opening. TG2 secretion is unaffected by blocking pannexin-1 channels. (C) P2X7R itself can form a membrane pore through conformational changes and, possibly, receptor oligomerization in a process that involves the extended intracellular C-terminal sequence. TG2 secretion is associated with this membrane pore activity but independent of ion channel function, and occurs in conjunction with thioredoxin-1 (Trx) externalization. As thioredoxin can reactivate TG2 functionally blocked in an oxidized state, this might ensure that externalized TG2 has transamidation activity. Flot2, flotillin-2.

A mutation in mouse P2X7R renders it deficient in pore-forming activity (Sorge et al., 2012). As the affected sequence motif in the P2X7R C-terminal domain is conserved in humans, we generated cells expressing human P2X7R with the analogous mutation P451L (Fig. 7B,C). However, these cells formed membrane pores in response to BzATP as revealed by YO-PRO1 uptake (Fig. 7D). This led us to investigate the gain-of-function P2X7R variant, A348T, which confers increased risk for autoimmune disease in man (Stokes et al., 2010), to substantiate a link between pore formation and TG2 secretion. Cells expressing P2X7R A348T (Fig. 7B,C) had a substantially increased propensity to form membrane pores as evidenced by enhanced peak pore activity (Fig. 7D) and by pore formation at very low BzATP concentrations (Fig. 7E). This enhanced pore activity was reflected in a corresponding increase in TG2 export (Fig. 7F,G). Interestingly, we also observed BzATP-induced secretion of thioredoxin-1 (Fig. 7H), an enzyme that can re-activate oxidatively inactivated TG2. This not only indicates that membrane pore activity controls the rate of TG2 export but that it leads to co-secretion of TG2 with thioredoxin-1 (Fig. 8).

## DISCUSSION

Here, we identify P2X7R as the central regulator of the pathway that enables active export of TG2 and its co-activator, thioredoxin-1. The action of both of these enzymes has been linked to specific immune responses (Iismaa et al., 2009; Jaeger et al., 2013), and this might therefore constitute a pathway for export of proteins relevant to innate immunity. Besides having roles in re-instating tissue integrity following injury or roles associated with infection control, TGs including TG2 have been implicated in immune regulation (Tóth et al., 2009; Loof et al., 2011). Here, we show that in monocytes and macrophages, purinergic signaling triggered rapid TG2 export in the absence of TLR engagement, and that this response was dependent on P2X7R but did not require caspase-1 activity. Likewise, introduction of P2X7R in HEK293 cells devoid of other inflammasome components (Lu et al., 2012) instated agonist-regulated rapid TG2 export. Taken together, the data demonstrate that P2X7R signaling alone is sufficient to trigger TG2 export, and involvement of an inflammasome-independent mechanism of export is further supported by the fact that externalized TG2 was not associated with vesicles or co-secreted with exosome-associated HMGB-1.

A redox-sensitive Cys switch promotes oxidative inactivation of TG2 (Stamnaes et al., 2010), a mechanism that is thought to contribute to rapid enzyme inactivation in the extracellular milieu (Jin et al., 2011) and thereby, to prevent aberrant crosslinking that might lead to fibrosis and potentially autoimmunity through neo-epitope formation (Aeschlimann and Thomazy, 2000; Iismaa et al., 2009). However, it has been shown that extracellular-matrix-associated TG2 can be reactivated by thioredoxin-1 released from activated monocytes during inflammation (Jin et al., 2011). Cell-surface-associated thioredoxin-1 plays a key role in innate immunity, particularly in mucosal epithelia where it activates β-defensin-1 (Jaeger et al., 2013). Interestingly, thioredoxin-1 is also an unconventionally secreted protein (Rubartelli et al., 1992), and our results show that it is in fact co-secreted with TG2. We speculate that thioredoxin-1 might not primarily act on pre-existing extracellular TG2 but could have a role as a chaperone during active TG2 export, enabling conversion of TG2 into its active conformation. Such a mechanism could explain why, in celiac disease, active TG2 accumulates in the intestinal mucosa (Korponay-Szabó et al., 2004).

### Purinergic signaling fulfills the pre-requisites for a unifying pathway regulating TG export

As TG2, and also other TGs, can be externalized by a range of cells including myeloid, mesenchymal, endothelial and epithelial cells (Aeschlimann and Thomazy, 2000; Nurminskaya and Belkin, 2012), it is implausible that this involves multiple highly divergent mechanisms as proposed previously. P2X7R is not restricted to the hematopoietic lineage as originally thought but is also widely distributed among mesenchymal, endothelial and epithelial cells, and in the central and peripheral nervous system (Bartlett et al., 2014). Activation of P2X7R occurs not only in conjunction with injury, cell stress and inflammatory processes but has major independent roles in the musculoskeletal (Garcia and Knight, 2010) and nervous system (Burnstock, 2015), contexts within which TG2-mediated extracellular reactions are also prevalent (Aeschlimann et al., 1995; Iismaa et al., 2009; Thomas et al., 2013).

Unlike previous work, our data implicate a regulated pathway in TG2 export. This mechanism might be activated to a varying extent under different conditions. A key finding here is that Ca^2+^ levels present in many media formulations impair TG2 release. Our data with catalytically inactive TG2 C277S show that this is not related to the regulation of TG2 by Ca^2+^ but due to suppressed P2X7R functionality, in line with evidence suggesting that divalent cations, including Ca^2+^, allosterically inhibit P2X7R (Yan et al., 2011). Therefore, the differences in extracellular Ca^2+^ or ATP concentrations might explain some contradictory findings in the literature. It is worth noting that modest shear stress during medium exchange or passaging can trigger cellular ATP release (Rumney et al., 2012) and, consequently, P2X7R-mediated TG2 release at low (0–1 mM) but not high (≥2 mM) [Ca^2+^]_ex_. Hence, endogenous P2X7R activation might explain apparently ‘constitutive’ TG2 secretion.

Crucially, in our HEK293 model, TG2 is not retained at the cell surface or internalized, unless an appropriate cell surface receptor is introduced (Fig. S4). Hence, TG2 export can be directly assessed by quantification in the cell supernatant. Thus, our system is overcoming intrinsic difficulties that hampered progress in the analysis of TG2 export previously, including quantification of cell-surface-associated TG2 without disrupting cell integrity or endocytic TG2 uptake and retrograde transport. This, together with modulation of the different P2X7R activities with small molecules or by mutagenesis, provided strong evidence for a direct link between P2X7R signaling and TG2 export. Our data is not contradictory to passive TG2 release as a consequence of a substantial insult, including mechanical damage (Upchurch et al., 1987) or TLR engagement (Siegel et al., 2008), or to vesicle-associated TG2 release under circumstances such as serum-starvation-associated cell stress (Antonyak et al., 2011). Rather, it suggests that purinergic signaling links controlled TG2 export to specific extracellular functions. Furthermore, given that microvesicle-associated TG2 has been shown to colocalize with fibronectin (Antonyak et al., 2011), an extracellular enzyme localization is implied. Therefore, it is possible that TG2 preferentially binds to plasma membrane subdomains where specific types of microvesicle form (pericellular matrix reorganization), thereby explaining the apparent association, but that this occurs subsequent to membrane translocation.

Different activities of TG2 as well as sequence motifs for interaction with proteins and phospholipids have been implicated in the export process (Balklava et al., 2002; Scarpellini et al., 2009; Chou et al., 2011; Zemskov et al., 2011). Our data show that transamidation activity is dispensable for export and that tagging TG2 with GFP does not prevent export, thereby excluding a terminal targeting signal. Blocking TG2 interaction with classically secreted proteins such as fibronectin, syndecans and integrins might alter extracellular localization or endocytic uptake and trafficking of TG2 (Antonyak et al., 2011; Chou et al., 2011; Zemskov et al., 2011) but cannot explain how the implied membrane translocation occurs.

### Mechanistically, TG2 export is linked to the secondary permeability pathway

Several lines of evidence show that TG2 export is linked to the ‘membrane pore’ activity associated with P2X7R activation (Fig. 8). Our data show that TG2 export is mechanistically separate from microvesicle shedding. In line with this, P2X7R induces bleb formation and microvesicle shedding through actin reorganization mediated by the MAPK p38 family and Rho activation, whereas YO-PRO1 uptake by cells is insensitive to cytochalasin-D (Pfeiffer et al., 2004). We further show that TG2 export is not induced by a [Ca^2+^]_i_ rise alone nor abrogated by pharmacological suppression of P2X7R ion channel function without affecting membrane pore formation. In contrast, introducing a mutation in P2X7R that enhanced pore activity resulted in accelerated TG2 export.

P2X7R is the only P2X receptor where membrane pore formation is consistently observed, and this activity is therefore a defining feature of it. Although mechanistically not fully understood, it requires the extended unique C-terminal intracellular tail (Smart et al., 2003; Sun et al., 2013). Recent data suggest that large cations can pass through the P2X7R channel itself, and that blocking the channel prevents dye uptake by cells (Browne et al., 2013). However, a larger channel diameter than expected from available structural data (Hattori and Gouaux, 2012) would be required to adequately explain permeation of some molecules, and a more substantial conformational change than predicted from existing structural data is indeed supported by a recent study (Allsopp and Evans, 2015). Interestingly, P2X7R also couples to effectors implicated in dye permeability, and a sustained [Ca^2+^]_i_ elevation by itself has been shown to trigger membrane pore opening (Bartlett et al., 2014). In our HEK293 model, calmidazolium attenuated Ca^2+^ influx but did not affect membrane pore activity suggesting that distinct permeation pathways are involved. Pannexin-1 is not involved, as shown with inhibitors, consistent with the data of others (Sun et al., 2013). Physiologically, the secondary permeability pathway might have a role in the release of secondary messengers, for example, glutamate release in P2X7R-expressing HEK293 cells has been reported (Cervetto et al., 2013). Given the delay between P2X7R-dependent Ca^2+^ signaling and detection of changes in extracellular TG2, we cannot exclude a role of a secondary messenger system. However, it is conceivable that this pathway constitutes a pore through which proteins can be trafficked through co-translocational unfolding (Rodriguez-Larrea and Bayley, 2014).

### TG2 activation is biological context-dependent

Given its requirement for high extracellular ATP concentration, P2X7R will primarily be activated after injury, in the context of inflammation or in the tumor microenvironment. Enhanced TG2 expression by resident fibroblasts and infiltrating myeloid cells is an integral part of the tissue repair response and leads to accumulation of extracellular TG2. TG2 secretion is thought to bring about its activation through Ca^2+^-induced conformational changes (Pinkas et al., 2007). However, it is possible that high extracellular nucleotide concentrations at sites of injury or inflammation not only activate P2X7R itself but also control TG2 activation as purine nucleotides are allosteric inhibitors, although the apparent binding affinity for ATP is low (∼1 mM) compared to GTP (∼3 µM) (Han et al., 2010; Thomas et al., 2013). Furthermore, a proposed heparan-sulfate-binding site is unique to the GTP-induced conformation (Lortat-Jacob et al., 2012) and such an interaction would stabilize this conformation and prevent Ca^2+^ binding. Therefore, it is worth noting that signaling functions for extracellular-nucleotide-bound enzyme have been postulated (Johnson and Terkeltaub, 2005; Tóth et al., 2009).

### Implications for TG2-mediated disease processes

P2X7R is highly polymorphic, and it has become increasingly clear that some amino acid mutations predispose to disease (Bartlett et al., 2014). We have shown here that a polymorphism in the second transmembrane domain that is associated with autoimmune disease (Stokes et al., 2010) facilitates membrane pore formation leading to enhanced TG2 secretion. This opens the possibility that the threshold for activation of TG2 export differs between individuals depending on their *P2RX7* genotype, and this might constitute a risk factor for diseases where TG2-mediated reactions cause pathology. This extends to animal models of disease. Notably, in contrast to mouse strain 129, the C57BL/6 background widely used in genetic studies carries P2X7R P451L which lacks the capacity to form membrane pores (Sorge et al., 2012). Different mouse lines might therefore differ with regards to the capacity for active TG2 export.

In conclusion, we have demonstrated that TG2 export is regulated by purinergic signaling, and that P2X7R plays a central role in this process. Our findings provide an explanation for the link between high levels of extracellular TG2 activity and inflammatory responses, and thereby identify a new avenue to limit TG2 activity therapeutically in conditions where enzyme function directly drives pathogenic processes, including fibrotic disease and gluten-related disorders.

## MATERIALS AND METHODS

### Cell culture

THP-1 monocytic leukemia cells were grown in suspension in RPMI1640 medium containing 10% heat-inactivated fetal bovine serum (FBS), streptomycin and penicillin. Mononuclear cells were isolated from heparinized human blood (with informed consent of donors and approval of the Research Ethics Committees, REC10/MRE09/28) on Ficoll-Plaque PREMIUM (GE Healthcare), washed in PBS, and cultured for 7 days as THP-1 cells but with addition of 20 ng/ml human GM-CSF (Preprotech) to derive M1 macrophages. HEK293 flp-in cells (Invitrogen) were cultured in Dulbecco's modified Eagle's medium (DMEM) containing 10% FBS, the above antibiotics and 100 µg/ml zeocin (Invitrogen). Experiments were conducted without antibiotics.

### Generation of stably transfected cell lines

P2X7R was amplified by PCR from image clone (ID:4298811) using the primers specified in Table S1 to generate wild-type, truncated and V5-tagged coding sequences, which were cloned into pcDNA5/V5-His/FRT vector (Invitrogen). Constructs for P2X7R mutants were generated by site-directed mutagenesis using the oligonucleotides given in Table S1. The coding sequence of all constructs was verified by sequencing. Cell lines were generated by co-transfection of P2X7R and recombinase (pOG44, Invitrogen) expression vectors into HEK293 flp-in cells using FuGENE 6 (Promega), followed by selection of stable transfectants with hygromycin B (100 µg/ml).

### THP-1 cell differentiation and activation

Cells were differentiated with 0.05 µg/ml TPA, and for IL-1β upregulation, treated with 100 ng/ml LPS for 24 h. For activation, cells (1×10^6^ cells/well) were suspended in PSS (10 mM Hepes-NaOH, pH 7.4, 147 mM NaCl, 2 mM KCl, 1 mM MgCl_2_, the indicated CaCl_2_ concentration and 12 mM glucose) and stimulated with ATP. Medium was carefully collected and rendered cell-free by centrifugation (1500 ***g***, 10 min). Cells were extracted on ice in 20 mM Hepes-NaOH, pH 7.4, 150 mM NaCl, 1 mM EGTA, 1% Triton X-100, 0.25% deoxycholate, 10% glycerol, 1 mM PMSF and 1 mM *N*-ethylmaleimide, and the extract was cleared by centrifugation (15,000 ***g***, 10 min, 4°C). IL-1β concentration in conditioned medium (100 µl) was determined by capture ELISA (Ready-SET-Go Set, eBioscience).

### Immunocytochemistry

Cells grown on poly-L-lysine-coated coverslips were fixed with 2% paraformaldehyde in PBS for 10 min, and permeabilized in 0.1% Triton X-100 in PBS. After blocking of non-specific binding with 1% BSA in PBS, P2X7R was detected with 2 µg/ml anti-P2X7R antibodies (sc-25698, Santa-Cruz Biotechnology) and Alexa-Fluor-488-conjugated secondary antibodies. Coverslips were mounted using Vectashield containing DAPI.

### [Ca^2+^]_i_ measurements in individual cells

Fluo-4-AM (Invitrogen) Ca^2+^ indicator was prepared in DMSO containing 20% Pluronic F-127. Cells (7×10^4^ cells/well) in poly-L-lysine-coated glass bottom dishes (50 mm; MatTek) were loaded for 20 min with 3 µM Fluo-4-AM in OptiMEM (Invitrogen). Medium was replaced with fresh OptiMEM, and cells were monitored by confocal microscopy during ATP or BzATP (Sigma) stimulation at 37°C and 5% CO_2_. Real-time videos were acquired (2.62 s between frames, 63× objective) using sequential scanning. For experiments with P2X7R antagonist, cells were loaded with Fluo-4-AM in OptiMEM containing 5 µM A740003 (Tocris) prior to stimulation with agonist in A740003-containing OptiMEM. Images were analyzed using the LAS-AF software (Leica).

### Analysis of TG2 externalization

Differentiated THP-1 cells (1×10^6^ cells/well) and primary macrophages (1×10^5^ cells/well, 24-well plates) were stimulated with P2X7R agonists in OptiMEM. HEK293 P2X7R or parental cells (1.5×10^5^ cells/well, 24-well plates) were transfected with 0.5 µg expression construct for wild-type TG2 or TG2 C277S (Stephens et al., 2004), or GFP-tagged TG2 (Table S2) using FuGENE-6. After 48 h, cells were washed with pre-warmed and gassed OptiMEM, and stimulated with P2X7R agonist or CPA (Merck-Millipore) in OptiMEM (250 µl/well). For inhibitor studies, cells were treated with 5 µM A740003, 1 µM calmidazolium chloride (Merck-Millipore), 10 µM BAPTA-AM (Merck-Millipore) or vehicle for 10 min, and then stimulated with BzATP in the presence of the respective inhibitors as indicated. Caspase-1 inhibitor Ac-YVAD-CMK was prepared fresh in OptiMEM and diluted to a 100 µM final concentration in experiments. Cell supernatant (pulse fraction) was collected, and cells washed with and subsequently incubated in pre-warmed and gassed OptiMEM without agonist for 30 min (chase fraction). Conditioned medium from four wells (six wells for macrophages) were combined, and rendered cell-free by centrifugation (1500 ***g***, 10 min) for analysis. Cell surface protein labeling with Sulfo-NHS-SS-biotin and purification was carried out with the Pierce cell surface protein isolation kit.

### Immunoblotting

Lyophilized (500 µl) or ethanol precipitated (1.3 ml, macrophages) conditioned media were reconstituted at 1/10th or 1/50th of the original volume in 12.5 mM Tris-HCl, pH 6.8, 4 M urea, 2% SDS, 20 mM EDTA, 2% β-mercaptoethanol and 15% glycerol. Protein concentrations of extracts were determined with a bicinchoninic acid protein assay. 20 µl reconstituted media or 10 µg cell extract together with Amersham LMW-SDS markers were separated on 4–20% SDS-PAGE Tris/glycine gels (Invitrogen) under reducing conditions, and transferred onto nitrocellulose membranes. For thioredoxin-1 detection, ethanol-precipitated (1:9, v/v) proteins (600 µl medium) were resuspended as above, and separated in 16% SDS-PAGE Tricine gels (Invitrogen) calibrated with Broad Range marker (11–190 kDa; NEB). Antibody labeling was performed as described previously (Aeschlimann et al., 1993) using the monoclonal antibodies CUB7402 against TG2 (0.2 µg/ml), TUB2.1 against β-tubulin (2.6 µg/ml), against flotillin-2 (0.5 µg/ml; 610383, BD-Biosciences), against HMGB-1 (0.73 µg/ml; ab184203, Abcam), against the V5tag (20 ng/ml) or the polyclonal anti-IκBα (1 µg/ml; sc-371, Santa Cruz Biotechnology), anti-caspase-3 (40 ng/ml; 9662, Cell Signaling), anti-P2X7R C-terminus (1 µg/ml) or anti-P2X7R extracellular domain (1.7 µg/ml; APR-008, Alomone Labs) antibodies. Anti-thioredoxin-1 antibodies (1:200; FL105, Santa Cruz Biotechnology) were used with 5% casein as blocking agent. Bound antibodies were detected with horseradish peroxidase (HRP)-conjugated secondary antibodies and Amersham ECL™ Plus/Prime. TG2 band intensity was quantified by densitometry using Image Lab 5.1 software (Bio-Rad).

### Analysis of cell damage and apoptosis

To assess cell integrity, LDH release was measured using CytoTox-ONE™ HMI Assay (Promega). Cells (1.2×10^5^ cells/well, 24-well plate) were treated with BzATP in 300 µl OptiMEM for 10 min (*n*=4), and cell-free conditioned media (100 µl) were analyzed for LDH. For estimation of total LDH, a replicate well set was subjected to cell lysis.

To assess whether treatment induced cell death, P2X7R cells were stimulated with BzATP and chased in OptiMEM as described above, and where indicated subsequently cultured in serum containing DMEM for up to 22 h. Cell extracts and particulate material recovered from conditioned media were analyzed for caspase-3 by immunoblotting.

### Localization of GFP-tagged TG2 using confocal microscopy

P2X7R cells on poly-L-lysine-coated coverslips were transfected with constructs for expression of N- or C-terminally GFP-tagged TG2. After 24 h, the coverslip was mounted for microscopy into a customized holder using silicone grease. Cells were kept in OptiMEM at 37°C and 5% CO_2_, and stimulated with a defined volume of agonist solution to obtain 1 mM ATP or 100 µM BzATP while monitoring GFP fluorescence and acquiring real-time movies.

### Detection and isolation of microvesicles

TG2- or mock-transfected P2X7R cells were stimulated with 100 µM BzATP or vehicle. Freshly collected conditioned media were rendered cell-free by centrifugation (1500 ***g***, 10 min) and supernatants analyzed for microvesicles by particle tracking using the NanoSight LM12 system with a high-sensitivity camera (Webber and Clayton, 2013). Five 60-s videos per sample (1×10^8^–5×10^8^ particles/ml) were recorded at 25.6 frames/s (gain=250), and analyzed using the NTA2.3 software. Alternatively, freshly collected conditioned media were subjected to differential centrifugation at 4°C, with 1500 ***g*** for 10 min, followed by 3000 ***g*** for 20 min, and then either 10,000 ***g*** for 30 min and 100,000 ***g*** (SW32Ti, Beckman) for 1 h or subjected to density gradient centrifugation. Supernatant (1.0 ml) was carefully layered on a Tris-buffered sucrose-step gradient (0, 20% and 60%) and centrifuged at 100,000 ***g*** for 90 min. Fractions (∼1 ml) constituting the top layer and 20%–60% interface (microvesicle fraction) as well as pellets were collected. Proteins were precipitated with 9 volumes of ethanol at 4°C, and analyzed using immunoblotting.

### P2X7R ‘membrane pore’ activity

Cells in poly-L-lysine-coated black optical 96-well plates (Nunc, 165305) were placed in PSS containing 0–2 mM Ca^2+^ and 1 µM YO-PRO1 (Invitrogen). The plate was transferred to a FLUOstar Omega reader (BMG Labtech) equilibrated to 37°C and 5% CO_2_. BzATP was injected to obtain a 0–500 µM final concentration (*n*=3) and fluorescence measured (4-mm orbital area) every 40 s for 30 min. Where indicated, cells were preincubated with 100 µM ^10^Panx (Tocris) for 10 min or 10–100 µg/ml trovafloxacin (Sigma) for 30 min and stimulated in the presence of inhibitors. After normalization for well-specific fluorescence, the average YO-PRO1 fluorescence of unstimulated cells was subtracted from that of agonist-stimulated cells to correct for bleaching. Dye uptake rates were derived by linear regression of data from the initial 5 min.

### [Ca^2+^]_i_ measurements in plate format

Cells (3×10^4^ cells/well) in optical 96-well plates were loaded with Fluo-4-AM, washed and placed in fresh OptiMEM (90 µl/well). After measuring baseline fluorescence, different concentrations of BzATP (0–300 µM) or medium alone were injected (10 µl/well). Fluorescence changes were measured in well mode over 20 s, with 40 0.1-s intervals followed by 0.4-s intervals. Data from eight wells per condition were averaged. The fluorescence of the control was subtracted from data with agonist treatment to correct for bleaching. Data (*F*, fluorescence; *t*, time) for the first 10 s were fitted using Eqn 1 to estimate the maximal fluorescence value (*F*_max_):
(1)
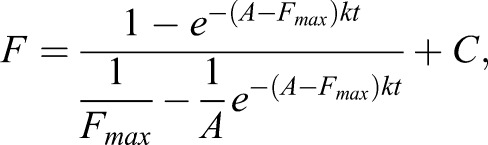
whereby *k* is the association constant, *A* is a function of agonist concentration and *C* is a constant for baseline correction. The association constant obtained from data fitting was *k*=1.8×10^−6^ M^−1^ s^−1^. *F*_max_ was then plotted against the agonist concentration to derive a dose–response curve.

### Membrane potential analysis

Voltage sensor probes, coumarin-labeled phospholipid CC2-DMPE (FRET donor) and oxonol dye DiSBAC_2_(3) (acceptor) were from Invitrogen. Cells (3×10^4^ cells/well) in optical 96-well plates were loaded with 10 µM CC2-DMPE in FRET buffer (10 mM Hepes-NaOH, pH 7.4, 160 mM NaCl, 0.9 mM CaCl_2_, 1 mM MgCl_2_, and 10 mM glucose) containing 200 µg/ml Pluronic F-127 for 30 min, washed and incubated in 100 µl 10 µM DiSBAC_2_(3) in FRET buffer for 20 min. Tartrazine (1.2 mM final concentration) was added, and after 10 min, fluorescence measurements (λ_ex_ of 420 nm; λ_em_ of 460 nm and 550 nm; 10-nm bandpass filters) were conducted in well mode at 37°C and 5% CO_2_. Gain was adjusted to yield similar baseline readings for each fluorophor at resting potential. Following baseline acquisition, 10 µl 0.82 M KCl, 1 mM BzATP or buffer control were injected while monitoring fluorescence. Following subtraction of signal without cells, the signal ratio (SR) before and at equilibrium after depolarization was calculated, and the response ratio (RR) derived as RR=SR^depol^/SR^pol^.

### Statistics

One-way ANOVA was used and significance between groups determined with Tukey's post-test, whereby *P*<0.05 was considered significant.
